# Molecular Mechanisms and Research Progress of Long Non-Coding RNAs in Regulating Mammalian Skeletal Muscle Development

**DOI:** 10.3390/genes17050592

**Published:** 2026-05-21

**Authors:** Xiaojiao Cui, Yongming Zhang, Ren Mu, Huimin Wei, Min Li, Xingdong Wang

**Affiliations:** 1 College of Life Science and Technology, Inner Mongolia Normal University, Hohhot 010022, China; 20244015047@mails.imnu.edu.cn (X.C.); zhangyongming@imnu.edu.cn (Y.Z.); murensk@imnu.edu.cn (R.M.); yisahm@163.com (H.W.); limin_8123@126.com (M.L.); 2 Key Laboratory of Biodiversity Conservation and Sustainable Utilization in Mongolian Plateau for College and University of Inner Mongolia Autonomous Region, Hohhot 010022, China

**Keywords:** long non-coding RNA, skeletal muscle development, muscle regeneration, gene regulation, CeRNA network controversy, myogenesis

## Abstract

Long non-coding RNAs (lncRNAs) have emerged as pivotal regulators in mammalian skeletal muscle development, moving beyond their initial characterization as transcriptional “noise”. Unlike previous reviews that focus primarily on individual IncRNA catalogues, this review systematically integrates recent advances across five dimensions: (1) molecular characteristics and multidimensional classification of muscle related lncRNAs; (2) stage-specific expression patterns spanning embryonic myogenesis, postnatal growth, adult maintenance, and regeneration; (3) underlying molecular mechanisms including chromatin remodeling, ceRNA networks, IncRNA protein interactions, and nucleocytoplasmic trafficking; (4) pathological implications in muscular dystrophy, atrophy, and neuromuscular diseases; (5) translational applications in precision animal breeding. We critically evaluate the controversial ceRNA hypothesis and highlight quantitative limitations in current evidence. By integrating existing knowledge into a multi-layer regulatory network model and addressing current technical challenges and controversies (e.g., the ceRNA stoichiometry debate), this review provides a comprehensive roadmap for future basic research and translational applications in muscle biology.

## 1. Introduction

Skeletal muscle development is a highly complex and precisely regulated biological process, which involves the differentiation of pluripotent mesenchymal precursor cells, the proliferation and fusion of myoblasts, and the formation of mature muscle fibers [1,2,3]. This process depends on the co-regulation of a variety of key transcription factors and signaling pathways [4]. (Paired box3) *Pax3* and (Paired box 7) *Pax7* are mainly expressed in myogenic precursor cells [5], while myogenic regulatory factors (MRFs) such as (Myogenic Differentiation 1) *MyoD*, *myogenin*, (Myogenic Factor 5) *Myf5* and (Muscle Regulatory Factor 4) *MRF4* dominate the differentiation and fusion of myoblasts [6].

In recent years, the discovery of long non-coding RNAs (lncRNAs), first described as a distinct class of regulatory molecules in the early 2000s [7], has provided new insights into the regulatory mechanisms of skeletal muscle development [8,9]. The first lncRNAs were described in the 1990s, including *H19* and *Xist*, which were initially identified through their imprinted expression patterns [10]. These lncRNAs, typically longer than 200 nucleotides, regulate gene expression at epigenetic, transcriptional, and post-transcriptional levels, playing important roles in chromatin modification, genomic imprinting, and pluripotency maintenance [11]. LncRNAs exhibit low sequence conservation across species but demonstrate significant functional conservation with tissue specificity [12]—a paradox that reflects their structural rather than sequence-based functional constraints [13]. At the epigenetic level, they can induce chromatin conformational changes at specific gene loci. For example, *DumlncRNA* recruits DNA methyltransferase (Dnmts) to the CpG site of Dppa2 promoter through chromatin internal circulation for methylation modification, resulting in silencing of *Dppa2* expression, thereby promoting myogenic differentiation [14]. However, whether chromatin remodeling represents the primary mechanism for all muscle lncRNAs remains unclear, as many nuclear lncRNAs may act through transcriptional scaffolding without direct chromatin modification [15]. LncRNAs such as *Braveheart* and *Fendrr* regulate the directional differentiation of cardiac lineages by recruiting polycomb repressor complex 2 (PRC2), and potentially analogous but not identical mechanisms may also be applicable to skeletal muscle development [16].

**Figure 1:**
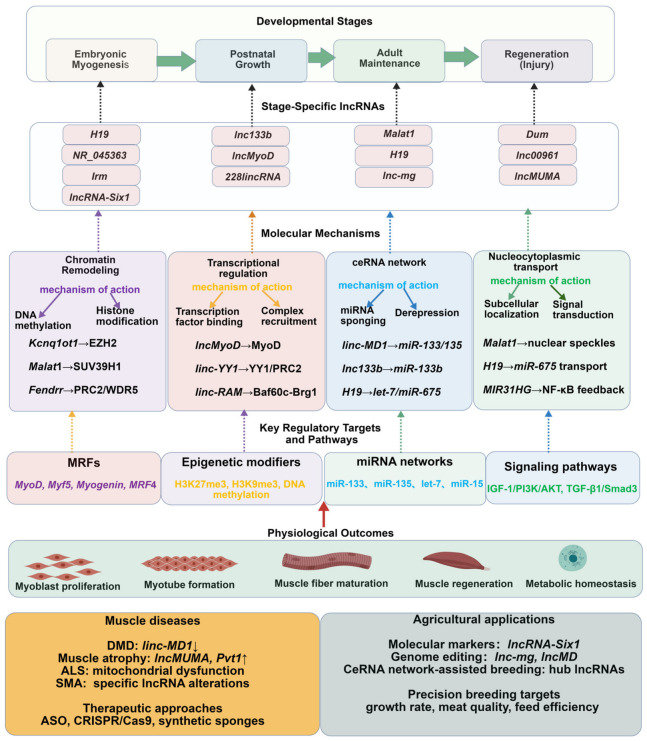
Regulatory flowchart of lncRNAs in skeletal muscle development.

LncRNAs play a central role in the regulation of skeletal muscle development [17], and the expression patterns of some lncRNAs show precise spatiotemporal specificity [17,18]. Studies have found that *lncRNA-1700113A16RIK* is present in skeletal muscle stem cells (MuSCs) and is significantly upregulated during differentiation. Functional verification shows that knockdown of this lncRNA inhibits the differentiation process of muscle stem cells, while overexpression promotes the differentiation process [19]. LncRNAs such as *MUNC* and *Dum* associated with *MyoD* have been identified as important regulators of muscle development [20]. Taking chicken skeletal muscle research as an example, among the 1995 skeletal muscle-related lncRNAs identified, molecules such as *lnc00003323* may regulate *TEAD4* expression through cis-acting, thereby affecting cell growth and proliferation [21]. Cross-species comparative studies reveal that while some lncRNAs (e.g., Malat1, H19) show conserved expression patterns, others are species-specific (e.g., lncRNA-Six1 in chicken), highlighting the need for cautious extrapolation across models [22,23,24]. Notably, the differential expression patterns of lncRNAs in different muscle types collectively highlight lncRNAs as pivotal regulators of skeletal muscle biology. However, the existing knowledge remains fragmented. The following sections deconstruct this model by detailing characteristic features, stage-specific functions, and underlying molecular mechanisms, with particular emphasis on implications for agricultural traits and muscular disorders. Despite these advances, several critical questions remain unanswered: (1) What is the relative contribution of chromatin remodeling versus ceRNA mechanisms to individual lncRNA functions? (2) How do lncRNAs maintain functional roles despite low sequence conservation—through conserved structural motifs or species-specific adaptations? (3) How can we move from single-lncRNA studies to a systems-level understanding of regulatory networks? (4) What are the quantitative thresholds for effective ceRNA sponging under physiological conditions? Figure 1 shows a regulatory flowchart of lncRNAs in skeletal muscle development. The diagram illustrates stage-specific lncRNA expression across four developmental phases (embryonic myogenesis, postnatal growth, adult maintenance, and regeneration following injury) and their integration into four core molecular mechanisms: chromatin remodeling, transcriptional regulation, ceRNA networks, and nucleocytoplasmic trafficking. These mechanisms converge on key regulatory targets including myogenic regulatory factors (MRFs: MyoD, Myf5, myogenin, MRF4), epigenetic modifiers (H3K27me3, H3K9me3, DNA methylation), miRNA networks (*miR-133*, *miR-135*, *let-7*, *miR-15*), and signaling pathways (IGF-1/PI3K/AKT, TGF-β1/Smad3), ultimately driving physiological outcomes such as myoblast proliferation, myotube formation, muscle fiber maturation, regeneration, and metabolic homeostasis. The lower panels highlight translational implications in muscle disorders (DMD, atrophy, ALS, SMA) and precision agriculture applications, including molecular marker-assisted selection, genome editing targets, and ceRNA network-assisted breeding strategies for improved growth rate, meat quality, and feed efficiency [25].

## 2. Functional Classification and Characteristics of lncRNAs

LncRNAs are a class of non-coding RNAs longer than 200 nt. They have a series of unique characteristics in molecular structure, which makes them significantly different from microRNAs, piwi-interacting RNAs (piRNAs) and other small RNA molecules [26]. In terms of evolutionary conservation, lncRNAs exhibit distinct characteristic patterns from protein-coding genes. Compared with protein-coding genes, lncRNAs are generally low in sequence conservation [27,28], but their functional conservation shows significant tissue specificity [29]. This apparent paradox may be explained by the predominance of structural over sequence constraints in lncRNA function [24,30]. Although most lncRNAs lack open reading frames and protein-coding functions, their complex secondary and tertiary structures allow them to act as regulators of key genes in skeletal muscle development [26,31]. Unlike protein-coding genes, many lncRNAs exhibit low sequence conservation, which may be related to their functional diversity [32]. In addition, lncRNAs are involved in the development and functional regulation of specific tissues and show high specificity in various tissues and cell types [33]. Moreover, the expression level of lncRNAs changes significantly during embryonic development and is strictly regulated by developmental timing [34].

### 2.1. Structural Characteristics

The length of lncRNAs varies significantly across different species and tissues [28]. For example, the average transcript length of lincRNAs in pig skeletal muscle is 776 bp, which is significantly lower than the known lincRNAs genes (1361 bp) and protein-coding genes (1828 bp) [35]. During the development of skeletal muscle in goats, the average length of lncRNA gene was 1296 bp, containing 2.4 exons, compared to the protein-coding gene (average length of 1978 bp, containing 8.4 exons) [36]. Similar patterns in cattle (1296 bp, 2.4 exons) [37] and rabbits (mostly two exons) [38] suggest conserved architectural features of muscle lncRNAs. Similar to mRNAs, lncRNAs are primarily transcribed by RNA polymerase II and typically possess a 5′ cap and a 3′ poly(A) tail [39]. Unlike mRNAs, they usually lack functional open reading frames (ORFs), and their protein-coding potential is extremely low or even completely missing [40]. This structural difference prevents lncRNAs from binding to ribosomes or encoding proteins [41]. Although most lncRNAs lack protein-coding ability, it is noteworthy that recent studies have found that some lncRNAs can encode small proteins, which enriches the understanding of their functions [42]. The secondary structure is an important molecular feature of lncRNAs. These molecules tend to form thermodynamically stable secondary and higher-order structures, which can be used as functional domains to participate in a variety of regulatory activities [30]. For example, in X chromosome-specific inactivated transcripts (*Xist*), repeat units A and C each shape unique secondary structures, which are critical for their recruitment of polycomb repressor complex 2 (PRC2) and bivalent protein YY1 [43]. *RoX1* and *roX2* lncRNAs in Drosophila contain a series of tandem stem-loop structures, and these structural changes directly affect the recruitment of male-specific lethal complexes (MSL) [44]. These examples indicate that the secondary structure of lncRNAs is closely related to their functions.

In addition, the genomic distribution of lncRNAs is diverse. They can be derived from introns (intron-type lncRNAs), coding exons, 3′ or 5′ untranslated regions (3′ or 5′ UTRs), and even overlap with their own transcripts in an antisense direction (natural antisense transcript, NAT) [45]. In the regulatory region, upstream of the promoter (promoter upstream transcript, PROMPT), enhancer (eRNA), intergenic region (lincRNA) and telomeres can produce lncRNAs [45]. This broad genomic distribution enables lncRNAs to participate in diverse regulatory networks. According to their location relative to protein-coding genes, lncRNAs can be divided into antisense, intergenic, bidirectional, intron and enhancer types [20]. The exon–intron structure of lncRNAs also exhibits unique properties. Compared with protein-coding genes, lncRNAs usually have fewer exons. In the study of lncRNAs related to rabbit skeletal muscle development, most lncRNAs contain only two exons [38]. In maize, 82.0% of lncRNAs contain no more than two exons, while the median number of exons of protein-coding genes is four [46]. This relatively simplified genetic architecture may be related to its functional diversity and regulatory flexibility [47]. It is worth noting that the exon length of lncRNAs is often longer than that of protein-coding genes. For example, the average transcript length of lincRNAs in pig skeletal muscle is 776 bp, which is significantly lower than the known lincRNAs genes (1361 bp) and protein-coding genes (1828 bp) [35]. During bovine skeletal muscle development, lncRNA transcripts exhibited an average length of 1296 bp and contained 2.4 exons on average, whereas protein-coding genes were longer, averaging 1978 bp with approximately 8.4 exons per transcript [37].

### 2.2. Functional Classification

The functional diversity of lncRNAs is closely related to their regulatory patterns and intracellular localization, which provides a foundation for establishing a classification framework [48]. Based on their regulatory mode, lncRNAs can be broadly categorized into two types: cis- and trans-acting. Cis-regulated lncRNAs usually play a role near their transcription sites and regulate the transcriptional activity of adjacent genes by attracting chromatin-modifying enzyme complexes or transcription factors [49]. For example, *Kcnq1ot1* regulates the expression of adjacent imprinted genes at the chromatin level through cis-acting [50], while *lnc00003323* may target *TEAD4* expression through a cis-acting mechanism [51]. Trans-regulated lncRNAs show a wider range of regulation and can play a role in distal loci. For example, *HOTAIR* inhibits the transcription of the *HOXD* gene cluster through trans-regulation [20]. This classification reflects differences in the spatial range of lncRNA action and highlights their distinct functional hierarchies within gene regulatory networks.

Long intergenic non-coding RNAs (lincRNAs) are transcribed from genomic regions between protein-coding genes and represent the largest class of lncRNAs—comprising ~96% of porcine lncRNAs [52]. Unlike genic lncRNAs, lincRNAs have independent transcriptional units and minimal overlap with protein-coding sequences, making them ideal candidates for trans-regulatory functions [53]. From the perspective of subcellular localization, lncRNAs can be further divided into two groups: nuclear enrichment and cytoplasmic enrichment. Intranuclear lncRNAs can be divided into cis-acting and trans-acting subtypes [54]. Among them, cis-acting lncRNAs mainly regulate gene expression by interacting with local chromatin, while trans-acting lncRNAs participate in long-distance regulation by constructing a three-dimensional chromatin structure or acting as scaffold molecules [55]. In the cytoplasm, lncRNAs play a role mainly by affecting mRNA stability, translation efficiency, or as miRNA sponges. Studies have shown that most lncRNAs are enriched in the cytoplasmic and ribosomal parts and are involved in regulating microRNA (miRNA) activity [56]. This subcellular distribution difference directly determines the molecular mechanism and functional characteristics of lncRNAs.

Based on genomic localization and transcription direction, lncRNAs can be further subdivided into a variety of structural subtypes. According to their relative position in the genome, they can be divided into sensing lncRNAs (derived from the same strand of protein-coding genes), anti-sensing lncRNAs (transcribed from the opposite strand of protein-coding genes), divergent lncRNAs (shared promoters with protein-coding genes), intron lncRNAs (completely embedded in the introns of protein-coding genes), and spacer lncRNAs (encoded by completely independent transcription units) [57]. Pig genome analysis showed that about 96% of lncRNAs were classified as inter-exon and intron lncRNAs, while only 4% were cis/trans lncRNAs [58]. This classification system not only helps to understand the origin and evolutionary relationship of lncRNAs, but also provides a framework for exploring their interaction with protein-coding genes.

From the perspective of functional mechanism, lncRNAs can also be classified according to their interaction patterns with chromatin. (Enhancer RNAs) ERNAs are a special class of lncRNAs, which are produced by transcription of enhancer regions and promote transcription initiation by forming chromatin loops and interacting with promoters [57]. Promoter-associated long RNAs (*PALRs*) overlap at the 5′ end of the protein coding region, containing the promoter region and the first exon or intron, which can recruit RNA-binding proteins to accurately regulate transcription dynamics [58]. This classification was first proposed by Kurokawa (2011) [59], distinguishing PALRs from other promoter-proximal transcripts by their specific protein-recruitment functions. In addition, lncRNAs can also act as signal molecules, bait molecules, guide molecules and scaffold molecules to participate in gene expression regulation. This functional classification was first proposed by Chang’s laboratory in 2011, which greatly promoted the development of lncRNA research [60].

Tissue-specific expression is another important classification standard for lncRNAs. Many lncRNAs exhibit strict spatio-temporal expression patterns, such as muscle-specific expression of *lncRNA linc-MD1*, which plays a key role in myoblast differentiation [61]. Further studies showed that lncRNAs showed dynamic expression characteristics at different stages of skeletal muscle development. For example, of the 462 differentially expressed circular RNAs (circRNAs) found during chicken embryonic development, *circRBFOX2* promotes cell proliferation by interacting with *miR-206* [62]. This spatio-temporal specific expression pattern enables lncRNAs to accurately regulate various key aspects of muscle development, covering all stages from stem cell fate determination to myotube formation and maturation. Table 1 summarizes the multi-dimensional classification system of the above lncRNAs, including key features such as mechanism of action, subcellular localization, genomic localization, functional mechanism and tissue-specific expression, which provides a structured framework for understanding the molecular regulatory network of lncRNAs.

## 3. Functional Roles and Molecular Mechanisms of lncRNAs in Skeletal Muscle Development

Skeletal muscle growth and development constitute complex processes requiring multi-cell type coordination, encompassing myoblast proliferation, migration, differentiation and myotube fusion [65]. From embryonic myogenesis to adult regeneration, the normal progress of this process depends on the synergy of various cell types, signaling pathways and transcription factors [1]. MRFs include *Myf5*, *MyoD*, *Myogenin* and *MRF4*. *Myf5*, as the earliest expressed muscle regulatory factor, plays an important role in the proliferation and differentiation of early muscle progenitor cells [66]. *MyoD* promotes the transformation of muscle progenitor cells into myofibrillar cells by cooperating with *Myf5* [66,67]. Subsequently, during the differentiation and maturation of myofibrillar cells, Myogenin promotes myotube formation [68]. In the late stage of muscle development, MRF4 plays an important role in the maturation and stability of muscle fibers [69]. While MRFs collectively drive myogenesis, individual lncRNAs preferentially target specific MRFs: LncMyoD and MUNC regulate MyoD [70], while Irm modulates MEF2D-MyoD complexes [71]. Table 2 summarizes the representative lncRNAs at different developmental stages and their expression characteristics, regulatory mechanisms and functional associations.

### 3.1. Stage-Specific Expression Patterns During Development

LncRNAs exhibit highly dynamic expression characteristics during the skeletal muscle development, which is directly related to their regulatory functions in myogenesis. Genome-wide analysis identified 577 differentially expressed lncRNA transcripts during goat skeletal muscle development, and functional studies confirmed their primary role in early myogenesis [36]. The study of rabbit muscle development also revealed the stage-specific expression characteristics of lncRNAs and mRNAs. RNA sequencing data showed that the samples at 0 days, 35 days and 84 days after birth showed obvious stage-specific expression patterns, which together confirmed that lncRNAs were involved in the construction of skeletal muscle development through precise temporal expression and dynamic regulatory networks [38]. During embryonic development, lncRNAs exhibit the most active and diverse expression patterns. For example, *NR_045363* is highly expressed in the embryonic stage, and the expression level decreases sharply in adulthood, suggesting that it may be involved in cell cycle regulation [72]. Similarly, *lncRNAIrm* is highly expressed in limb muscles of newborn mice, but gradually decreases with development [73]. In addition, 1995 lncRNAs related to skeletal muscle development were identified in the study of embryonic development of Xinghua chickens, some of which regulated the expression of adjacent genes through a cis-acting mechanism and affected cell growth and proliferation [74]. The regulatory network of lncRNAs in postnatal development is more complex. The expression level of *lnc133b* in bovine skeletal muscle satellite cells increased gradually during the development process and peaked under dexamethasone (Dex) conditions [75]. *LncMyoD* is hardly expressed in resting state, but it is significantly upregulated within two days after muscle injury, and its expression is also detected in muscle stem cells differentiated in vitro, indicating that this molecule may be involved in the muscle regeneration process [76].

In adult skeletal muscle, *H19* is abundantly expressed in embryonic skeletal muscle and maintains significant expression in adult muscle, suggesting functional conservation in muscle development and structural maintenance—not myogenesis per se, as myogenic activity is minimal in adult muscle under homeostatic conditions [77]. The expression of *Malat1* was significantly increased during myoblast differentiation and participated in myogenesis through the *miR-181a-Malat1-MyoD/Suv39h1* regulatory axis; during cell proliferation, *Malat1* recruited *Suv39h1* to the *MyoD* binding site to induce *H3K9me3* modification to inhibit target genes, and during differentiation, *miR-181a* degrades nuclear *Malat1* transcripts through an Ago2-dependent mechanism [78]. Cross-species comparative studies further revealed the conservation and specificity of lncRNAs expression patterns. For example, 228 potential lincRNAs were identified in porcine skeletal muscle development studies, most of which were differentially expressed at different developmental stages [23]. LncRNA-*Six1* in chicken muscle can not only cis-regulate *Six1* gene expression, but also encode a micropeptide with a molecular weight of about 7260, which directly affects muscle cell proliferation [79]. These findings together describe the complex regulatory network constructed by lncRNAs in skeletal muscle development, and its stage-specific expression provides important clues for analyzing the molecular mechanism of muscle development. Cross-study comparisons reveal notable discrepancies in reported expression patterns. For example, while H19 is constitutively described as highly expressed in embryonic muscle, its adult expression level varies across reports—possibly due to differences in muscle type (limb vs. diaphragm), species, or the presence of regenerative stimuli. Such inconsistencies highlight the need for standardized sampling and developmental staging in future studies.

**Table 2:** Representative lncRNAs and their functional characteristics across different developmental stages.

Developmental Stage	Representative lncRNAs	Expression Profile	Regulatory Mechanism	Functional Association
Embryonic Stage	*H19*	Highly expressed during embryogenesis, gradually declines thereafter [80].	Regulated by transcription factors such as *MyoD* and *MEF2* [80].	Involved in cell cycle regulation [72].
	*NR_045363*	Significantly upregulated in embryos, sharply decreases in adulthood [72].	Modulated by epigenetic modifications [11].	Influences cell growth and proliferation [81].
	*Irm*	Highly expressed in neonatal mouse limb muscle, decreases during development [73].	Directly binds *MEF2D* to modulate *MyoD/MEF2D* transcriptional activity [73].	Promotes myogenin and *miR-206* expression [73].
Postnatal Stage	*lnc133b*	Expression increases progressively during development, peaking under dexamethasone (Dex) conditions [75].	Regulated by DNA methylation and histone modifications [75].	Affects skeletal muscle satellite cell function [75].
	*lncMyoD*	Barely detectable in quiescent state, significantly upregulated within two days post-injury [76].	Binds IGF2-mRNA-binding protein 2 (IMP2) to inhibit translation of proliferation genes [76].	Promotes myoblast exit from the proliferation cycle [76].
Adult Stage	*Malat1*	Expression significantly increases during myoblast differentiation [82].	Participates in myogenesis via the *miR-181a–Malat1–MyoD/Suv39h1* axis [82].	Suppresses target genes during proliferation and relieves suppression during differentiation [82].
	*H19*	Constitutively highly expressed in both embryonic and adult skeletal muscle [80].	Functionally conserved regulatory mechanisms [80].	Maintains myogenic regulatory function [80].
Cross-Species Comparison	*lncRNA-Six1*	Specifically expressed in domestic chicken muscle [22].	*Cis*-regulates *Six1* expression and encodes a micropeptide [22].	Directly influences muscle cell proliferation [22].
	*228 lincRNAs*	Identified in porcine skeletal muscle development, mostly differentially expressed across stages [23].	Species-specific regulatory networks [23].	Participates in muscle development regulation [23].

### 3.2. Underlying Molecular Mechanisms

**Figure 2:**
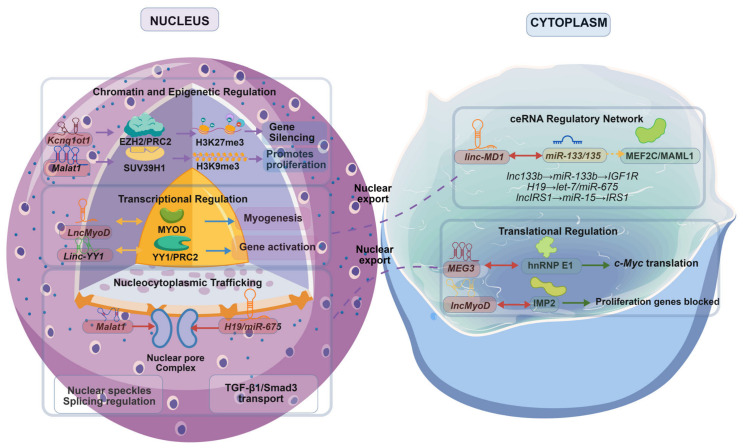
Molecular mechanisms of lncRNA action in muscle cells.

In Figure 2, we summarize the diverse molecular mechanisms through which lncRNAs exert regulatory functions in muscle cells, encompassing both nuclear and cytoplasmic action modes. Figure 2: Molecular mechanisms of lncRNA action in muscle cells. (Left) Nuclear mechanisms: (i) Chromatin and epigenetic regulation—*Kcnq1ot1* recruits EZH2/PRC2 to induce H3K27me3-mediated gene silencing, while *Malat1* recruits SUV39H1 to establish H3K9me3, promoting proliferation; (ii) transcriptional regulation—*lncMyoD* binds MyoD to drive myogenesis, and *linc-YY1* interacts with YY1/PRC2 to activate gene expression; (iii) nucleocytoplasmic trafficking—*Malat1* localizes to nuclear speckles to regulate splicing, and *H19/miR-675* modulates TGF-β1/Smad3 nuclear transport. Dashed arrows indicate nuclear export. (Right) Cytoplasmic mechanisms: (i) ceRNA regulatory network—*linc-MD1* sponges *miR-133/135* to derepress MEF2C and MAML1; additional examples include *lnc133b → miR-133b* → *IGF1R*, *H19 → let-7/miR-675*, and *lncIRS1* → *miR-15* → *IRS1*; (ii) translational regulation—*MEG3* binds hnRNP E1 to suppress *c-Myc* translation, and *lncMyoD* sequesters IMP2 to block proliferation gene expression. The bottom panel summarizes methodological validation approaches: ChIP-seq/ATAC-seq for chromatin remodeling, CLIP-seq/eCLIP for RNA-protein interactions, RIP-seq for protein binding, single-molecule FISH for colocalization, CRISPRi/a for functional screening, and snRNA-seq for cell-type specificity [25].

#### 3.2.1. Chromatin Remodeling and Epigenetic Regulation

Chromatin remodeling represents one of the most prevalent mechanisms through which lncRNAs control stage-specific gene expression during myogenesis, particularly in stem cell fate determination and myotube formation [83]. As a key molecule in the regulation of myogenic gene expression, the mechanism of lncRNAs is mainly reflected in the interaction with chromatin modification complexes [39]. Studies have found that lncRNAs can specifically recognize histone methyltransferases or demethylases, or guide chromatin modification complexes to specific genomic regions through spatial localization, so as to achieve fine regulation of target gene transcriptional activity [84]. Taking *Kcnq1ot1* as an example, this molecule acts as a molecular scaffold in undifferentiated muscle cells, mediating the binding of *EZH2* to *p57Kip2*, promoting the enrichment of H3K27me3 at the *p57Kip2* site, and eventually leading to the inhibition of *p57* gene expression and myogenic differentiation [85]. This finding provides important evidence for elucidating the epigenetic regulation function of lncRNAs in muscle development. Studies have shown that *Malat1* can specifically recruit *Suv39h1* to the *MyoD* binding site, induce the formation of H3K9me3 modification, and then inhibit the expression of related genes [78]. *Fendrr* promotes the establishment of H3K27me3 modification by enhancing the binding ability of PRC2 in the promoter region, and achieves continuous regulation of target gene expression [86]. *Dum* lncRNA recruits Dnmts to the CpG site of the *Dppa2* promoter region through the chromatin internal circulation mechanism, inducing methylation modification, resulting in silencing of *Dppa2* expression, thereby promoting myogenic differentiation [87]. *HOTTIP* specifically activates the transcription of the *HOXA* gene cluster by directly interacting with the WDR5/MLL complex [88]. This bidirectional regulatory feature enables lncRNAs to precisely balance pro- and anti-differentiation genes during muscle development. The epigenetic functions of lncRNAs described above have been largely uncovered using chromatin immunoprecipitation coupled with sequencing (ChIP-seq) and assay for transposase-accessible chromatin with sequencing (ATAC-seq). ChIP-seq enables genome-wide mapping of histone modifications (e.g., H3K27me3, H3K9me3) and transcription factor binding sites, allowing researchers to determine whether a lncRNA recruits repressive or activating complexes to specific genomic loci [89]. However, standard ChIP-seq cannot distinguish direct lncRNA-recruited modifications from indirect effects secondary to transcriptional changes; temporal ChIP-seq (e.g., after inducible lncRNA knockdown) and ChIP-reChIP are needed to establish causality. ATAC-seq assesses chromatin accessibility, which can reveal how lncRNAs such as *LncMyoD* modulate the open state of enhancer or promoter regions [76]. Together, these techniques provide critical evidence for lncRNA-mediated chromatin remodeling during myogenesis. Recent methodological innovations have transformed lncRNA functional validation. CRISPRi/a screening in human iPSC-derived myotubes identified 47 essential muscle lncRNAs with fitness scores, overcoming immortalized cell line artifacts [90]. Single-nucleus RNA-seq (snRNA-seq) of human muscle biopsies resolved lncRNA expression across 12 distinct cell types, revealing that 60% of bulk-detected “muscle lncRNAs” are actually fibroblast or endothelial contaminants [91]. Spatial transcriptomics mapped lncRNA-MALAT1 to regenerating myofibers with 2 μm resolution, confirming its absence from quiescent satellite cells [92]. For protein interaction validation, eCLIP-seq with improved UV crosslinking efficiency achieved single-nucleotide resolution of LncMyoD-MyoD binding at the bHLH domain [93]. These advances address prior limitations but introduce new challenges: CRISPRi/a requires lentiviral delivery with potential insertional mutagenesis; snRNA-seq loses cytoplasmic lncRNAs during nuclear isolation; and spatial transcriptomics remains limited to fresh-frozen tissues, excluding archived biopsy collections.

#### 3.2.2. CeRNA-Mediated Regulatory Networks

The competitive endogenous RNA (ceRNA) mechanism allows lncRNAs to fine-tune the post-transcriptional availability of miRNAs, thereby modulating the expression of key myogenic factors during myoblast differentiation and muscle fiber type specification. However, this hypothesis faces significant quantitative challenges. Stoichiometric analyses suggest that lncRNA:miRNA binding ratios are often insufficient for effective sponging under physiological conditions [94]. Most ceRNA studies rely on overexpression systems, and rigorous validation using endogenous knock-in of miRNA binding site mutations remains rare [95]. Furthermore, compensatory mechanisms—where multiple lncRNAs target the same miRNA or miRNA families regulate overlapping target sets—may buffer against single lncRNA perturbations [96]. As ceRNAs, lncRNAs play a key regulatory role in skeletal muscle development. Studies have shown that lncRNAs can sequester microRNAs through a sponging effect, thereby removing the inhibitory effect of miRNAs on the expression of target gene mRNAs [97]. *Linc-MD1* acts as a molecular sponge of *miR-133* and *miR-135*, and can block the inhibitory effect of these miRNAs on their target mRNAs (including *MAML1* and *MEF2C*) [98]. *lnc133b* enhances the expression of *IGF1R* by adsorbing *miR-133b*, thereby promoting the proliferation and differentiation of skeletal muscle satellite cells [75]. *LncMUMA* reduces the inhibitory effect of *miR-762* on *MyoD* by competitively binding to *miR-762* with *MyoD*, and ultimately promotes myogenic differentiation [20]. *H19* contains let-7 binding sites and also produces *miR-675-3p* and *miR-675-5p*, which are highly expressed in skeletal muscle and are upregulated during myoblast differentiation and muscle regeneration [6,99]. *MyHC-IIA/X-AS*, as a lncRNA with ceRNA function, maintains the expression of *MyHC-IIx* and fast muscle fiber phenotype by sponge effect on *miR-130b* [80]. In the study of chicken skeletal muscle development, *lncIRS1* regulates the expression of *IRS1* gene by recruiting *miR-15* family and activates the expression of genes related to IGF-1 signaling pathway, thus promoting muscle fiber hypertrophy and alleviating muscle atrophy [100]. Supporting evidence for ceRNA function in muscle includes: (1) phenotypic rescue upon lncRNA restoration in DMD models [101]; (2) correlative expression patterns during differentiation [102]; (3) CLIP-seq confirmation of direct miRNA–lncRNA binding [103]. These findings establish the universality and importance of ceRNA networks in muscle development. While the ceRNA hypothesis remains influential, recent quantitative studies have challenged its physiological relevance. In 2024, Huang et al. demonstrated that functionally conserved lncRNAs from zebrafish to human maintain regulatory roles despite <50% sequence homology, suggesting structural rather than sequence-based functional constraints [24]. The stoichiometric problem persists: most muscle lncRNAs are expressed at low copy numbers (10–100 molecules per cell), whereas target miRNAs often exceed 1000 copies, making effective sponging mathematically improbable under physiological conditions [104]. Single-molecule RNA imaging in human primary myoblasts confirmed that lncRNA–miRNA colocalization is rare, occurring in <5% of cells under basal conditions [105]. Furthermore, CRISPR-mediated knock-in of miRNA binding site mutations in endogenous lncMD failed to phenocopy the overexpression studies, suggesting that observed effects may reflect non-specific RNA overexpression artifacts rather than genuine ceRNA function [106]. These findings necessitate a balanced evaluation: while ceRNA networks exist in vitro, their in vivo significance in muscle development remains unproven [95]. Most ceRNA studies rely on overexpression systems, and rigorous validation using endogenous knock-in of miRNA binding site mutations remains rare [95]. Future work must address the quantitative threshold question: under what stoichiometric conditions (lncRNA:miRNA:target mRNA ratios) does sponging become physiologically significant?

#### 3.2.3. lncRNA–Protein Interactions

Direct binding of lncRNAs to transcription factors, co-regulators, and RNA-binding proteins constitutes a core mechanism for fine-tuning transcriptional programs during muscle stem cell differentiation and regeneration. Crosslinking immunoprecipitation (CLIP-seq) and variants (eCLIP, HITS-CLIP) map RNA-binding protein (RBP) binding sites transcriptome-wide. RIP-qPCR validates specific interactions, while RIP-seq enables unbiased identification. These approaches discovered *lncMyoD–MyoD* and *Irm–MEF2D* interactions central to myogenic regulation. *LncMyoD* can selectively recognize MyoD protein and form a stable transcriptional activation complex with strict site specificity; each functional domain of MyoD (N-terminus, bHLH, C-terminus) can independently mediate this binding, suggesting multivalent binding [76]. By specifically recognizing MEF2D protein, *Irm lncRNA* can accurately locate the *MyoD/MEF2D* transcription complex to the promoter region of the target gene and significantly enhance its transcriptional activation potential [73]. As a target of *MyoD* transcriptional regulation, *linc-RAM* promotes the formation of the MyoD-Baf60c-Brg1 complex by interacting with MyoD protein, thereby exerting an enhancer-like regulatory function in the promoter region of myogenic genes [107]. In addition, *linc-YY1* specifically binds to the transcription factor YY1 to remove the YY1/PRC2 from the target promoter region, thereby activating downstream gene expression [108]. *Fendrr* can combine PRC2 and WDR5 to dynamically regulate gene expression by coordinating histone modification status [109]. *MEG3* affects the translation of *c-Myc* mRNA by interacting with hnRNP E1, demonstrating the diversity of lncRNAs regulating gene expression through protein–RNA interaction networks [11]. *Neat1* regulates myoblasts through a dual pattern: downregulating *P21* to promote proliferation while inhibiting myogenic marker gene transcription to delay differentiation [80]. From an evolutionary perspective, lncRNA–protein interactions not only retain conserved core modules, but also show species specificity. Studies have found that about 20% of mammalian lncRNAs can bind to the PRC2 complex [110]; EZH2 as a catalytic subunit inhibits target gene transcription by inducing H3K27 methylation. LncRNAs such as *HoxA-AS3* and *ANCR* have been found to induce H3K27 methylation by specifically binding to EZH2, thereby inhibiting the expression of key myogenic regulatory factors such as *Runx2* [111]. These phenomena suggest that lncRNA–protein interactions may maintain a basic regulatory framework during evolution and evolve diverse regulatory networks in different species. The identification and validation of lncRNA–protein interactions rely heavily on crosslinking immunoprecipitation followed by CLIP-seq and its variants (e.g., eCLIP, HITS-CLIP), which map RNA-binding protein (RBP) binding sites on lncRNAs transcriptome-wide. For hypothesis-driven studies, RNA immunoprecipitation (RIP) followed by quantitative RIP-qPCR is widely used to validate specific lncRNA–protein interactions, while RIP-seq enables unbiased identification of all RNAs associated with a given protein [112,113]. These approaches have been instrumental in discovering interactions like *Ln–MyoD–MyoD* and Irm–MEF2D, which are central to myogenic regulation [73,76].

#### 3.2.4. Regulation of Nucleocytoplasmic Trafficking

The subcellular localization of lncRNAs—whether nuclear or cytoplasmic—determines their mode of action and enables them to influence critical processes such as cell cycle progression, signaling pathway activation, and myogenic gene expression [48]. LncRNAs are important regulatory factors in nucleoplasmic transport. They affect the transport of proteins and RNA across the nuclear membrane through various mechanisms [114]. These mechanisms include regulating the function of nuclear pore complexes. They also include changing the activity of transporters. Additionally, they include participating in the assembly of transporters as molecular scaffolds [114]. Taking *Malat1* lncRNA as an example, its nuclear localization in muscle cells is closely related to cell cycle regulation [115]. This molecule is primarily localized to nuclear speckles—dynamic subnuclear compartments that serve as storage and assembly hubs for RNA processing and gene regulatory components. Through interaction with SR family splicing factors, it regulates alternative splicing of pre-mRNAs and thereby influences the expression of synapse-related genes [116]. It is worth noting that *Malat1* can also inhibit *MyoD*-mediated gene activation by recruiting Suv39h1 inhibitory complex to *MyoD* binding locus, thereby regulating muscle differentiation [117]. For example, *miR-675-3p* and *miR-675-5p* encoded by lncRNA *H19* can form a feedback regulation loop to regulate the nuclear transport process of the TGF-β1/Smad3 signaling pathway [118]. In the inflammatory response, after the activation of the NF-κB pathway, the nuclear LncRNA-*MIR31 HG* will be transported to the cytoplasm, and the NF-κB subunit P65 directly binds to its promoter region to enhance transcriptional activity, forming a positive feedback loop [119]. The downregulation of *LncRNA-ANCR* leads to a decrease in the expression of *GSK3β*, which promotes the nuclear transport of β-catenin by inhibiting its degradation, thereby enhancing the activity of the Runx2 receptor and promoting the osteogenic differentiation of mesenchymal stem cells [120]. The specific binding of *SRA* lncRNA to SRAP protein can block SRA-mediated regulation of *MyoD* transcriptional activity, indicating that lncRNA–protein interaction plays an important role in nucleoplasmic transport [115].

## 4. Roles of lncRNAs: From Disease Mechanisms to Agricultural Applications

### 4.1. lncRNAs in Muscle Disorders: Mechanisms of Action

Recent studies have revealed that aberrant expression and dysfunction of lncRNAs play key roles in the pathogenesis of various muscle diseases [121]. Especially in muscle diseases represented by muscular dystrophy and muscle atrophy, the expression levels of specific lncRNAs such as *Malat1*, *Linc-YY1* and *Dum* are significantly changed. These molecules participate in the disease process by regulating the expression of muscle development-related genes [122]. Table 3 summarizes the evidence that some lncRNAs are involved in muscle pathophysiological processes and evaluates their potential as potential therapeutic targets.

The mechanism of action of lncRNAs in muscle diseases has become a research hotspot. While cardiac hypertrophy involves lncRNA dysregulation (e.g., *CTBP1-AS2* stabilizing *TLR4* mRNA) [123], this review focuses on skeletal muscle diseases. Cardiac and skeletal muscle share some lncRNA regulators (e.g., *Malat1*, *H19*) but exhibit distinct pathophysiological mechanisms. Readers are referred to cardiac-specific reviews for detailed discussion of *CTBP1-AS2* [124]. Decreased expression of *Linc-MD1* lncRNA was observed in patients with DMD, and experiments confirmed that restoring the expression of this lncRNA can significantly improve the differentiation ability of DMD cells [125]. The molecular mechanisms of lncRNAs involved in muscle diseases are significantly diverse. *Linc-MD1*, as a typical representative, regulates the expression levels of MEF2C and Mastermind-like protein 1 by competitively binding to *miR-133* and *miR-135*, thereby affecting myoblast differentiation [126]. In addition to the ceRNA mechanism, lncRNAs can also mediate epigenetic regulation. *Malat1* lncRNA recruits *Suv39h1* to *MyoD* binding sites in proliferating myoblasts, induces H3K9me3 modification and inhibits target gene expression [127]. These findings provide a new perspective for understanding the molecular basis of muscle diseases.

Abnormal expression patterns of lncRNAs were also observed in neuromuscular diseases. In the model of amyotrophic lateral sclerosis (ALS), *Pvt1* is upregulated in atrophic conditions and modulates *c-Myc* stability, affecting Bcl-2, *Bax/Bak*, *Mfn1*, and *Beclin 1* expression, thereby impacting mitochondrial function, autophagy, and apoptosis [128]. This change leads to mitochondrial dysfunction and abnormal apoptosis, eventually leading to muscle fiber atrophy. Changes in the expression of specific lncRNAs have also been detected in patients with spinal muscular atrophy (SMA) [129]. Knockdown of *Pvt1* enhances resistance to muscle atrophy. *LncIRS1* sequesters the *miR-15* family to regulate *IRS1* expression, activating *IGF-1* signaling to promote muscle fiber hypertrophy and alleviate atrophy progression [100]. *LncMuMA* inhibits unloading-induced downregulation of *MyoD*, maintaining muscle mass, cross-sectional area, and function; its overexpression ameliorates muscle atrophy [130]. Similarly, the expression level of *lnc-mg* is positively correlated with myogenic ability, and its knockout leads to muscle atrophy, while overexpression can increase muscle mass [131]. While the therapeutic potential of these lncRNAs is exciting, the majority of findings are based on rodent models or immortalized cell lines [132]. Human muscle biopsy data are scarce, with only three studies profiling lncRNAs in DMD patient biopsies [125,133,134]. Future studies should prioritize validation in human primary myoblasts or organoids.

In recent years, the application value of lncRNAs in the diagnosis of muscle diseases has gradually emerged. Genome studies have shown that a large number of disease-related single nucleotide polymorphisms (SNPs) are concentrated in intergenic regions rich in non-coding RNA, which provides a theoretical basis for lncRNAs as diagnostic markers [135]. Studies on cardiovascular diseases have shown that the analysis of lncRNAs from body fluids has become a new idea for early detection of diseases [136]. This strategy is also applicable to the diagnosis of muscle diseases. When muscle tissue is damaged or diseased, specific lncRNAs will enter the peripheral circulatory system [137]. Clinical observations of muscle atrophy diseases have found that the expression fluctuations of some lncRNAs are significantly correlated with disease progression, which creates conditions for the development of non-invasive diagnostic methods [138]. Studies at the molecular level have revealed that *lncRNA199592* and *HC* regulate lipid metabolism through the *hnRNPA2B1*-mediated mRNA degradation mechanism, which may provide new biomarkers for the diagnosis of metabolic muscle diseases [139]. While the therapeutic potential of these lncRNAs is exciting, the majority of findings are based on rodent models or immortalized cell lines. Human muscle biopsy data are scarce Future studies should prioritize validation in human primary myoblasts or organoids.

**Table 3:** Roles of lncRNAs in muscle-related diseases and their therapeutic potential.

Disease Type	lncRNA Name	Expression Change	Regulatory Mechanism	Functional Impact	Therapeutic Potential
Muscular Dystrophy	*Linc-MD1*	Downregulated	Functions as a ceRNA for *miR-133* and *miR-135* to regulate *MEF2C* and *MAML1* expression	Restores differentiation capacity in DMD myoblasts	Overexpression ameliorates differentiation defects [115,140]
	*Dum*	-	Recruits DNMT1/3a/3b to mediate Dppa2 promoter methylation	Regulates myogenic differentiation progression	Potential therapeutic target [115]
	*LncMyoD*	-	Competitively binds IMPs	Regulates cell cycle exit and differentiation [141]	-
Muscle Atrophy	*lncMuMA*	-	Inhibits unloading-induced downregulation of *MyoD*	Maintains muscle mass, cross-sectional area, and function	Overexpression ameliorates muscle atrophy [131]
	*Pvt1*	Upregulated	Modulates *c-Myc* stability, affecting *Bcl-2*, *Bax/Bak*, *Mfn1*, and *Beclin 1* expression	Impacts mitochondrial function, autophagy, and apoptosis	Knockdown enhances resistance to muscle atrophy [56]
	*lncIRS1*	-	Sequesters the *miR-15* family to regulate *IRS1* expression	Activates IGF-1 signaling to promote muscle fiber hypertrophy	Alleviates muscle atrophy progression [100]
	*lnc-mg*	-	-	Maintains muscle structure and function	Overexpression reduces muscle mass loss [17]
Muscle Injury Repair	*Irm*	-	Modulates *MyoD/MEF2D* transcriptional activity	Promotes expression of myogenic markers (myogenin and MHC)	Overexpression enhances differentiation potential [73]
	*DUM*	Upregulated	-	Improves muscle regeneration efficiency	Potential therapeutic target
	*Malat1*	-	Participates in myogenesis via the *miR-181a–Malat1–MyoD/Suv39h1* axis	Knockout enhances muscle regeneration post-acute injury [142]	
	*Lnc00961*	-	Encodes the micropeptide SPAR that inhibits mTORC1 activity	Regulates the muscle regeneration process post-injury	-

### 4.2. Diagnostic Biomarkers and Therapeutic Targets

In addition to diagnostic value, lncRNAs have also become a new target of concern in the field of disease treatment. It has been found that the expression of *CHAST* lncRNA is abnormally increased during cardiac hypertrophy, and inhibition of its expression by antisense oligonucleotide (ASO) technology can significantly improve the pathological process of heart failure model [143]. The mechanism of *Pvt1* lncRNA in muscle atrophy has also been elucidated [56]. Its downregulation can not only protect oxidized (Type I) muscle fibers from denervation-induced atrophy, but also upregulate *Mfn1* expression, thereby improving mitochondrial function network [56]. These findings lay a theoretical foundation for the development of innovative therapies for muscle degenerative diseases.

ASO-mediated knockdown, CRISPR/Cas9-based disruption, and small molecule inhibitors targeting lncRNA–protein interactions are under development [144]. For instance, ASOs targeting *Malat1* have shown efficacy in preclinical models [145]. The ceRNA network also offers opportunities: delivering synthetic sponges or using CRISPRa to upregulate endogenous ceRNA lncRNAs may rewire regulatory circuits in diseased muscle [146]. Delivery specificity to skeletal muscle, potential off-target effects, and the low sequence conservation of lncRNAs across species remain hurdles for clinical translation [147]. Nonetheless, the tissue-specific expression patterns of many muscle-related lncRNAs provide a window for targeted therapies.

### 4.3. Translational Applications in Animal Breeding and Precision Agriculture

The functional dissection of lncRNAs in farm animals (e.g., pigs, chickens, cattle, and goats) opens transformative avenues for genetic improvement [148]. Moving beyond traditional protein-coding genes, lncRNAs offer a new dimension for precision breeding.

Established applications: Muscle-specific or highly expressed lncRNAs, such as the porcine lincRNAs identified across developmental stages [149] and the chicken *lncRNA-Six1* [150], can serve as valuable molecular markers for genomic selection. Genetic variations (e.g., SNPs or structural variants) within these functional lncRNA loci could be associated with muscle growth rate, lean meat percentage, or feed efficiency [151]. Incorporating such markers into breeding value estimation models could enhance the accuracy of selection for complex traits.

Speculative but promising strategies: Functionally validated lncRNAs present novel targets for genome editing. For instance, CRISPR/Cas9-mediated enhancement of *lnc-mg* or *lncMD* expression, which promotes myogenesis via the *miR-125b/IGF2* axis [7], could be a strategy to boost muscle mass. Conversely, fine-tuning the expression of lncRNAs like *MyHC-IIA/X-AS*, a key regulator of fast-twitch fiber identity [152], offers a direct path to modulating meat quality traits such as tenderness and water-holding capacity, which are critically important for consumer satisfaction and product value.

Finally, the ceRNA network theory provides a blueprint for network-based breeding strategies [153]. Instead of targeting single genes, modulating a key “hub” lncRNA (e.g., *linc-MD1*) could coordinately rewire an entire regulatory network affecting muscle differentiation and hypertrophy [154]. This systems-level approach, which we term “ceRNA Network-Assisted Breeding,” may lead to more robust and sustainable genetic gains by leveraging inherent biological buffering systems.

## 5. Prospects, Future Directions, and Limitations

### 5.1. Current Limitations

This review has several limitations. First, the ceRNA mechanism, while extensively discussed, lacks rigorous quantitative validation in muscle systems—most evidence comes from overexpression studies with limited endogenous validation [155]. Second, species differences are substantial: human–mouse lncRNA sequence homology is <80%, complicating translational extrapolation [156]. Third, claims about therapeutic and breeding applications are sometimes speculative; we have distinguished established findings from future possibilities throughout [157]. Fourth, single-cell resolution is lacking in most studies, masking cell-type-specific lncRNA functions [158]. Fifth, although preliminary progress in human biopsy data has been made—for example, single-nucleus RNA-seq of muscle biopsies from DMD patients has revealed marked increases in inflammatory/immune responses and extracellular matrix remodeling [159], and SNP analyses together with whole blood gene expression profiling have opened new avenues for liquid biopsy applications in spinal muscular atrophy (SMA) [160]—the majority of findings derive from rodent models or immortalized cell lines [161]. Primary myoblast studies from DMD patients still indicate an urgent need for direct validation in human samples [162], and transcriptomic data from human muscle biopsies remain limited and leave a substantial gap [163].

### 5.2. Future Research Directions

Although significant progress has been made in understanding the roles of lncRNAs in regulating skeletal muscle development, several key challenges remain. At present, the functional verification of lncRNAs faces technical bottlenecks, and its molecular mechanism of action, including participating in the regulation as a miRNA sponge and affecting chromatin remodeling, still lacks more detailed and direct experimental support [164]. In addition, there are significant obstacles in cross-species research. The fact that the homology of human and mouse lncRNAs sequences is less than 80% suggests that we need to be cautious about the conservation differences between species [156]. On the other hand, the association mechanism between tissue-specific expression patterns and functional diversity of lncRNAs during muscle stem cell fate determination and muscle fiber type transformation is still unclear [165]. In response to the above challenges, future research needs to focus on the development and application of new functional verification technologies. Innovative methods such as improved CRISPR screening system (e.g., CRISPRi/a for non-coding regions) and single-cell spatio-temporal transcriptome analysis are expected to break through the limitations of existing technologies [166]. The rapid development and wide application of high-throughput sequencing technology are driving the research field of lncRNAs to expand to multiple dimensions [167]. The introduction of spatial transcriptomics technology has brought a new perspective for lncRNAs research [168]. This technology can accurately capture the temporal and spatial expression characteristics of lncRNAs in skeletal muscle tissues, and provides an important tool for elucidating their functional mechanisms at different stages of muscle development. In the field of animal husbandry, lncRNA research has opened up a new way to improve livestock muscle growth traits and meat quality. Future research should also strengthen the integration of multi-omics data and apply artificial intelligence prediction methods to more systematically analyze the core mechanism of lncRNAs in the regulation network of skeletal muscle development.

### 5.3. Conclusions

This review synthesizes current understanding of lncRNA regulation in skeletal muscle development, emphasizing mechanistic diversity, stage-specific expression, and translational potential. We highlight the need for quantitative validation of ceRNA hypotheses, standardized developmental staging, and cross-species functional conservation studies. Future research should prioritize: (1) endogenous validation using knock-in models; (2) single-cell resolution of lncRNA expression dynamics; (3) integration of structural and sequence conservation analyses; and (4) rigorous preclinical testing in human primary myoblasts or organoids before therapeutic application.

## Figures and Tables

**Table 1:** Classification and functional characteristics of lncRNAs in skeletal muscle development.

Classification Criteria	Subtype	Main Characteristics	Representative Examples and Functions
Mode of Action	Cis-acting lncRNAs	Function near their transcription sites by recruiting chromatin-modifying complexes or transcription factors to regulate adjacent gene expression [63].	*Kcnq1ot1* regulates the expression of nearby imprinted genes; *lnc00003323* targets *TEAD4* expression [50].
	Trans-acting lncRNAs	Function at distal genomic loci, participating in long-range regulation by facilitating 3D chromatin structure formation or acting as molecular scaffolds [20].	*HOTAIR* represses the transcription of the *HOXD* gene cluster [20].
Subcellular Localization	Nuclear-enriched lncRNAs	Include *cis*- and *trans*-acting subtypes; participate in local chromatin interactions or long-range regulation [17].	*Cis*-acting lncRNAs regulate local gene expression; *trans*-acting lncRNAs mediate chromatin structuring [17].
	Cytoplasmic-enriched lncRNAs	Influence mRNA stability, translation efficiency, or act as miRNA sponges; primarily enriched in the cytoplasm and ribosomal fractions [56].	Regulate miRNA activity [56].
Genomic Context	lincRNAs	Located in genomic regions between protein-coding genes; comprise ~96% of porcine lncRNAs [58].	May interact with protein-coding genes [58].
	Intronic lncRNAs	Collectively account for ~96% of porcine lncRNAs [58].	May interact with protein-coding genes [58].
	Sense/Antisense lncRNAs	Transcribed from independent transcriptional units in sense or antisense orientation; comprise ~4% of porcine lncRNAs [58].	May interact with protein-coding genes [58].
Functional Mechanism	eRNAs	Transcribed from enhancer regions; facilitate transcription initiation by forming chromatin loops with promoters [64].	Involved in the regulation of transcription initiation [64].
	*PALRs*	Overlap the 5′ end of protein-coding regions; recruit RNA-binding proteins to modulate transcription [58].	Contain promoter sequences and the first exon or intron [58].
Tissue-Specific Expression	Muscle-specific lncRNAs	Exhibit dynamic expression patterns across different stages of skeletal muscle development [34,38].	*linc-MD1* is crucial for myoblast differentiation; *circRBFOX2* interacts with *miR-206* to promote proliferation [34,38].

## Data Availability

Data availability is not applicable to this article as no new data were created or analyzed in this study.

## Abbreviations

The following abbreviations are used in this manuscript:

**Abbreviations**	**Expanded Form**
ALS	Amyotrophic lateral sclerosis
Pax3	Paired box 3
Pax7	Paired box 7
MyoD	Myogenic Differentiation 1
Myf5	Myogenic Factor 5
MRF4	Muscle Regulatory Factor 4
MRFs	Myogenic regulatory factors
Dex	Dexamethasone
ASOs	Antisense oligonucleotides
CeRNA	Competitive endogenous RNA
circRNAs	Circular RNAs
DMD	Duchenne muscular dystrophy
Dnmts	DNA methyltransferases
eRNAs	Enhancer RNAs
HC	Hepatic Cholesterol Regulator
IMP2	IGF2-mRNA-binding protein 2
lincRNAs	Long intergenic non-coding RNAs
lncRNAs	Long non-coding RNAs
miRNA	MicroRNA
MPCs	Muscle progenitor cells
MSL	Male Specific Lethal
MuSCs	Muscle stem cells
NATs	Natural antisense transcripts
ORFs	Open reading frames
PALRs	Promoter-associated long RNAs
piRNAs	Piwi-interacting RNAs
PRC2	Polycomb Repressive Complex 2
RBPs	RNA-binding proteins
RNP	Ribonucleoprotein
SMA	Spinal muscular atrophy
SNPs	Single nucleotide polymorphisms
UTRs	Untranslated regions
Xist	X-inactive specific transcript
